# Jasmonic acid levels decline in advance of the transition to the adult phase in maize

**DOI:** 10.1002/pld3.180

**Published:** 2019-11-26

**Authors:** Krista Osadchuk, Chi‐Lien Cheng, Erin E. Irish

**Affiliations:** ^1^ Department of Biology University of Iowa Iowa City IA USA

**Keywords:** adult, jasmonic acid, juvenile, LC‐MS, maize

## Abstract

Leaf‐derived signals drive the development of the shoot, eventually leading to flowering. In maize, transcripts of genes that facilitate jasmonic acid (JA) signaling are more abundant in juvenile compared to adult leaf primordia; exogenous application of JA both extends the juvenile phase and delays the decline in miR156 levels. To test the hypothesis that JA promotes juvenility, we measured JA and meJA levels using LC‐MS in successive stages of leaf one development and in later leaves at stages leading up to phase change in both normal maize and phase change mutants. We concurrently measured gibberellic acid (GA), required for the timely transition to the adult phase. Jasmonic acid levels increased from germination through leaf one differentiation, declining in later formed leaves as the shoot approached phase change. In contrast, levels of GA were low in leaf one after germination and increased as the shoot matured to the adult phase. Multiple doses of exogenous JA resulted in the production of as many as three additional juvenile leaves. We analyzed two transcript expression datasets to investigate when gene regulation by miR156 begins in the context of spatiotemporal patterns of JA and GA signaling. Quantifying these hormones in phase change mutants provided insight into how these two hormones control phase‐specific patterns of differentiation. We conclude that the hormone JA is a leaf‐provisioned signal that influences the duration, and possibly the initiation, of the juvenile phase of maize by controlling patterns of differentiation in successive leaf primordia.

## 1 INTRODUCTION

During shoot ontogeny, leaves provide a visual display of current developmental status as well as the cues that direct future developmental processes. Leaf‐derived signals also trigger systemic changes in gene expression in response to abiotic stress (Karpinski et al., 1999; Miller et al., 2009; Mühlenbock et al., 2008; Mullineaux, Karpinski, & Baker, 2006; Rossel et al., 2007; Szechyńska‐Hebda, Kruk, Górecka, Karpińska, & Karpiński, 2010) and herbivory (Farmer & Ryan, 1992; Seo et al., 2001; Li, Li, Lee, & Howe, 2002; reviewed by Arimura, Ozawa, & Maffei, 2011). During the reiterative process of leaf initiation, expansion, and differentiation, the shoot progresses through different developmental phases: the juvenile phase is followed by the adult vegetative phase and then the reproductive phase, during which flowering and fruit formation occurs. The mechanism by which leaves of photoperiodic plants produce Flowering Locus T, a phloem‐mobile signal that evokes flowering (Huang, Böhlenius, Eriksson, Parcy, & Nilsson, 2005), has been thoroughly analyzed. Manipulation of several plant species established that signals from leaf tissue also affect the phase identity of the shoot earlier, during vegetative growth. Experiments in *Arabidopsis* suggest that sugar is a leaf‐derived signal that promotes vegetative phase change (Yang, Xu, Koo, He, & Poethig, 2013; Yu et al., 2013), while leaf‐produced miR156 serves to maintain juvenility throughout the young shoot (Fouracre & Poethig, 2019). The non‐cell autonomy of both *Teopod1* and *Teopod2*, dominant mutations that prolong the juvenile phase in maize, suggested the existence of a diffusible factor that regulates juvenile development in that species (Dudley & Poethig, 1993; Li et al., 2018; Poethig, 1988). Likewise, whether an isolated maize shoot apex is rejuvenated in culture depends on how many leaf primordia remain attached (Irish & Karlen, 1998; Orkwiszewski & Poethig, 2000), implicating a leaf‐derived signal in directing adult phase identity.

Underlying the juvenile and adult vegetative phases is the dynamic expression of two microRNAs. The expression of miR156 is high during the juvenile phase, declining as the adult phase is achieved, as first described in *Arabidopsis* (Wu & Poethig, 2006; Yang, Conway, & Poethig, 2011) and subsequently found in many other species including maize (Beydler, Osadchuk, Cheng, Manak, & Irish, 2016; Zhang et al., 2009). miR156 prevents the expression of SPL transcription factors that direct flower development (Preston & Hileman, 2013) and is both necessary and sufficient for the juvenile phase in *Arabidopsis* (Wu et al., 2009). *Corngrass* mutants of maize exhibit an extended juvenile phase as a result of overexpression of miR156 (Chuck, Cigan, Saeteurn, & Hake, 2007). miR172 is high during the adult phase (Zhang et al., 2009) when it targets transcripts of AP2‐type transcription factors, including the floral repressing TOE of *Arabidopsis* (Zhang, Wang, Zeng, Zhang, & Ma, 2015) as well as those responsible for juvenile leaf characteristics in maize (Lauter, Kampani, Carlson, Goebel, & Moose, 2005; Moose & Sisco, 1996). The ability of miR156 to maintain the juvenile phase depends on expression above a threshold level (He et al., 2018); however, how high levels of miR156 are achieved to begin the juvenile phase is still not fully understood.

Our goals in this study were twofold. First, we quantified endogenous levels of the hormone jasmonic acid (JA) and the volatile and phloem‐mobile, methylated derivative, meJA, in maize at selected stages from germination to phase change, and in phase change mutants. We sought to confirm high levels of JA in juvenile maize leaf primordia indicated by previous gene expression studies (Beydler et al., 2016). We monitored levels of meJA to explore the possibility that it is a leaf‐derived signal that imposes juvenility throughout the young shoot: long‐distance systemic JA signaling is achieved by its methylated form (Seo et al., 2001). Since gibberellic acid (GA) has been shown to promote vegetative phase change (Evans & Poethig, 1995) we also quantified the bioactive form GA_3_ at the same developmental time points in normal maize and in phase change mutants in order to investigate how JA and GA might counteract each other to influence the vegetative phase of the shoot. Second, we explored maize transcript expression patterns from dry seed through seedling establishment and sections of developing juvenile leaf three to uncover the signaling dynamics of GA and JA in the context of leaf phase identity acquisition.

## 2 RESULTS

### 2.1 Endogenous JA and meJA levels are high in differentiating juvenile leaf tissue

**Figure 1:**
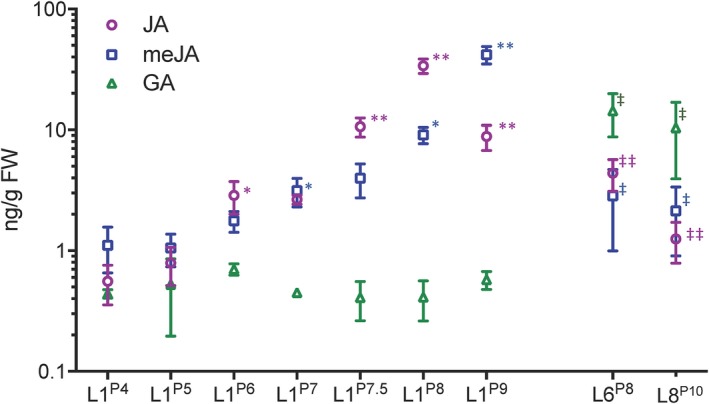
Endogenous concentration of JA, meJA, and GA in mo17xB73 maize hybrid of leaf 1 at successive developmental time points, transition leaf 6 at plastochron 8, and adult leaf 8 at plastochron 10. Values are shown as means of three to six biological replicates. gFW, grams fresh weight. * indicates significant difference of previous developmental stage using unpaired students *t* test *p* < .05, ** indicates *p* < .01. ^‡^ indicates significant difference compared with L1^P8^, *p* < .05. ^‡‡^ indicates *p* < .01

A previous comparison of transcript profiles of juvenile versus adult leaf primordia showed an enrichment of transcripts involved in relief of oxidative stress and in JA signaling in juvenile leaves (Beydler et al., 2016). Moreover, transcripts for JA methyl transferase (JMT), the enzyme that converts JA to the mobile, methylated derivative (meJA) involved in systemic signaling, were both highest in leaf one primordia (Beydler et al., 2016) and increased as leaf one develops. These results suggest that JA and meJA levels are high in juvenile leaves (the first four in the Mo17/B73 hybrid used here) compared to adult leaves. We used LC‐MS to quantify JA and meJA in leaf one at plastochrons four to nine (P4–P9), which range from 2 to 90 mm in length (Table S1). Since leaf differentiation occurs basipetally, each developing leaf primordium displays a gradient of developmental stages; to focus on the most mature tissue characteristics at each successive plastochron, only the terminal 10 mm was sampled from any primordium longer than 20 mm. In leaf one, we found increasing levels of JA as the leaf developed, with a highest level in P8 tips and slightly lower level in P9 (Figure 1, Table S2), the stage by which the tip appears to be fully differentiated. The levels of JA we observed are consistent with the range of JA levels previously reported in whole maize seedlings (Engelberth, Seidl‐Adams, Schultz, & Tumlinson, 2007; Yan et al., 2012) and tassels (Acosta et al., 2009) as well as during the response to wounding in maize leaves (Yan et al., 2012). Levels of meJA followed a similar but delayed increase during leaf one differentiation from P4 to P8. At P9, when JA levels had begun to decline, meJA was at its maximum level, matching the pattern of JMT transcript accumulation. Accordingly, we chose leaf one at P8 (L1^P8^) as a benchmark for JA levels in developing juvenile leaves.

**Figure 2:**
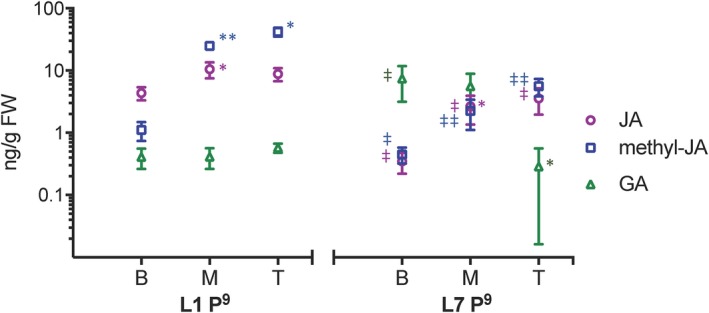
Endogenous concentration of meJA and JA in juvenile leaf 1 (left) and transition leaf 7 (right) at plastochron 9 (approximately 80 mm in length) of mo17xB73 maize hybrid at the bottom (B), middle (M), and top (T) 10 mm of the leaf blade. Values were calculated from means of *n* = 3. JA and methyl‐JA concentration is higher in leaf 1 than leaf 7, and in both leaves, JA concentration is higher at the tip of the leaf compared to the base. Leaf 1 GA levels are low throughout the blade, and leaf 7 GA levels are higher than in leaf 1 with the highest levels measure at the base of the leaf. *Y*‐axis scale is Log_10,_ and values are shown as means of three biological replicates. gFW, grams fresh weight. * indicates significant difference between adjacent leaf sections using unpaired students *t* test *p* < .05, ** indicates *p* < .01. ^‡^ indicates significant difference of leaf section between leaf 1 and leaf 7 using unpaired students *t* test *p* < .05. ^‡‡^ indicates *p* < .01

If high levels of JA and meJA are characteristic of the juvenile phase in maize, we expected levels to decrease as the shoot undergoes vegetative phase change. To test this, we measured levels in developing transition leaves six and seven and in leaf eight, the first fully adult leaf. As a transition leaf, fully expanded leaf six has juvenile traits such as epicuticular wax at the tip and adult traits such as trichomes at the base, but at P8 the leaf is still pale green and undifferentiated, just beginning to show juvenile phase‐specific morphological traits at its tip. Jasmonic acid levels in L6^P8^ were approximately eight times lower compared to L1^P8^ and meJA levels were three times lower (Figure 1, Table S2). Leaf seven had similarly lower levels of JA and meJA compared to leaf one (Figure 2, Table S2). Because highest JA levels were found in leaf one when it was almost full length and subsequent leaves attain lengths substantially greater than leaf one, leaf eight was sampled at the more advanced stage of P10. Nonetheless, L8^P10^ showed considerably lower levels of both JA and meJA: JA levels were approximately 32 times higher in L1^P8^ (and four times higher in L6^P8^) compared to levels in L8^P10^. meJA levels were four and a half times higher in L1^P8^ than L8^P10^, and more than 20 times higher in L1^P9^ than in L8^P10^ (Figure 1, Table S2). Together, these results demonstrate that while JA and meJA levels increase within a leaf as it grows, levels at comparable stages are significantly lower in later formed, developing adult leaves compared to juvenile leaves.

The patterns of JA and meJA accumulation in leaf one are consistent with that leaf producing the hormone and then modifying it for systemic distribution. It was of interest to determine whether the entire leaf or only a specific region/developmental zone is involved in systemic signaling. Comparisons of JA and meJA levels in L1^P9^, the stage with the highest levels of meJA, at the basal, middle, and tipmost 10 mm of tissue revealed that levels of JA in L1^P9^ were twice as high at the tip and middle compared to the base, while meJA levels were twice as high in the tip compared to the middle and barely detectable in the base (Figure 2, Table S2). As maize leaves show a basipetal pattern of differentiation, this pattern corresponds to highest JA and meJA in the most developmentally advanced regions of leaf one. To determine whether higher JA and meJA levels at the more differentiated leaf tip is specific to juvenile leaves, we compared leaf one levels to those of transition leaf seven, which would at maturity display juvenile traits at the tip and adult traits at the base. The highest levels of meJA were measured at the tip and decreased basipetally in L7^P9^, as was seen in L1^P9^; however, the level at the tip of the leaf was nine times higher in L1^P9^ compared to L7^P9^. Similarly, JA levels in L1^P9^ in the middle and tip were twice those in L7^P9^. Overall, levels of JA and meJA appeared to be highest in the terminal portion of the leaf, which is more differentiated, and lowest at the less differentiated base, mirroring the steady increase in JA and meJA measured in successive plastochrons of leaf one.

### 2.2 Maize phase change mutants display altered JA and meJA levels

**Figure 3:**
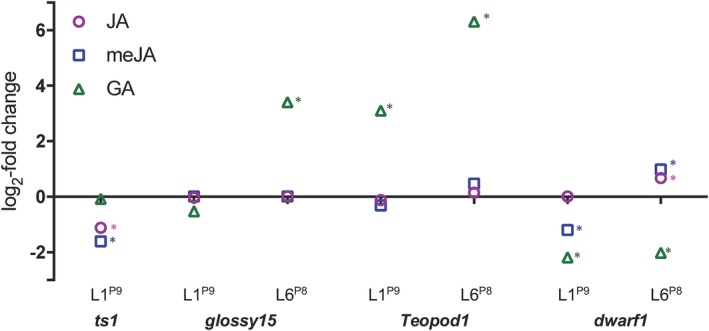
Log**_2_**‐fold change in concentration of JA, meJA, and GA of phase change mutants ts1, gl15, Tp1, dwarf1 compared to wild‐type siblings. Values were calculated from means of *n* = 3. Stars indicate significant difference using unpaired student's *t* test *p* < .05. Values were represented as log_2_‐fold change to ease visual comparison of hormone levels between mutants and wild‐type sibs at different developmental stages

A number of maize mutants display altered timing of vegetative phase change. For example, *glossy15* (*gl15*) and *tasselseed1* (*ts1*) have a truncated juvenile phase, in contrast to the GA‐deficient *dwarf* mutations, such as *dwarf* 1 (*d1*) or the dominant *Teopod1* (*Tp1*), which exhibit an extended juvenile phase by delaying the transition to adult. We compared levels of JA and meJA in phase change mutants to their normal sibs in L1^P9^, when meJA was highest, and in L6^P8^, representing the approximate stage when phase change has occurred in normal plants. Jasmonic acid‐deficient *tasselseed1 (ts1)* mutants are impaired in JA biosynthesis, which leads to feminized tassel florets (Acosta et al., 2009) and slightly early vegetative phase change (Beydler et al., 2016). While JA and meJA have been quantified in tassels of *ts1* (Acosta et al., 2009), JA levels in developing juvenile leaves have not been reported. As expected, JA and meJA levels were significantly lower in L1^P9^ of *ts1* compared to normal sibs (approx. two and three times lower, respectively) (Figure 3, Table S3). The AP2 transcription factor Glossy15, a target of the adult miR172, is required for normal distribution of epicuticular wax and lack of trichomes on maize leaves during the juvenile phase: *gl15* mutants exhibit early phase change and have only two, rather than four or five juvenile leaves (Evans & Poethig, 1995; Moose & Sisco, 1996). Levels of JA and meJA did not differ significantly between *gl15* mutants and normal sibs in L1^P9^ or in L6^P8^. *d1* mutants are characterized by an extended juvenile phase (Evans & Poethig, 1995) as well as reduced stature. Levels of JA were not different in L1^P9^ between *d1* mutants and their normal sibs, although levels of meJA were less than half that of normal in *d1* mutants. L6^P8^ of *d1* mutants*,* which are wholly juvenile when fully expanded, displayed significantly higher levels of both JA and meJA compared to L6^P8^ of their normal sibs, which are transition leaves displaying a mosaic of juvenile and adult characteristics. The dominant *Teopod1* (*Tp1)* mutation confers an exceptionally prolonged juvenile phase (Poethig, 1988). Although this gene has yet to be characterized, it has been shown to act non‐cell autonomously, suggesting that *Tp1* regulates a diffusible factor that influences shoot identity (Dudley & Poethig, 1993; Poethig, 1988). Jasmonic acid and meJA levels were not significantly different between mutants and their wild‐type siblings in either L1^P9^ or L6^P8^. Thus, it is unlikely that meJA is the diffusible factor responsible for prolonged juvenility of *Tp1* plants.

### 2.3 Jasmonic acid cannot delay phase change indefinitely in maize

**Figure 4:**
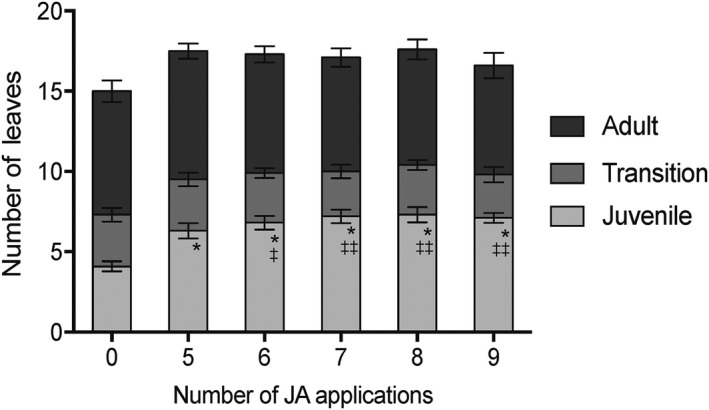
Effect of increasing number of doses of 5 mM JA on number of leaves with phase‐specific epidermal traits. Increasing number of doses increased the number of leaves with juvenile leaf characteristics but did not have an effect on the number of transition leaves or number of leaves with adult characteristics. Values are shows as mean of *n* = 10. * indicates significant difference of juvenile leaves compared with untreated plants using unpaired students *t* test *p* < .0001. Plants that received six through nine doses had a significantly different number of juvenile leaves than plants that received five doses, ^‡‡^ indicates *p* < .0005, ^‡^ indicates *p* < .05

We previously tested whether treatment with exogenous JA could affect phase change and found that a single dose of 15 mM JA both delayed expression of adult traits in transition leaves and led to an increase in levels of miR156 in leaf 5 (Beydler et al., 2016). Multiple treatments extended the juvenile phase in a dose‐dependent manner: five doses of 5 mM JA resulted in one additional wholly juvenile leaf (leaf 5) but did not change the number of transition or adult leaves (Beydler et al., 2016). To test the extent to which JA can delay vegetative phase change, we provided five to nine doses of 5 mM JA to seedlings starting a week after germination and treating every 48 hr. Adult traits were observed by the ninth leaf for all treated plants, even those that received nine applications of JA, with maximal extension of juvenility from seven doses (Figure 4). Thus, JA was unable to delay phase change indefinitely. Compared to control plants, there were at most three additional wholly juvenile leaves formed. In contrast, the number of transition and adult leaves was not changed in JA‐treated plants.

### 2.4 Juvenile phase transcriptome patterns are not acquired until after germination

**Figure 5:**
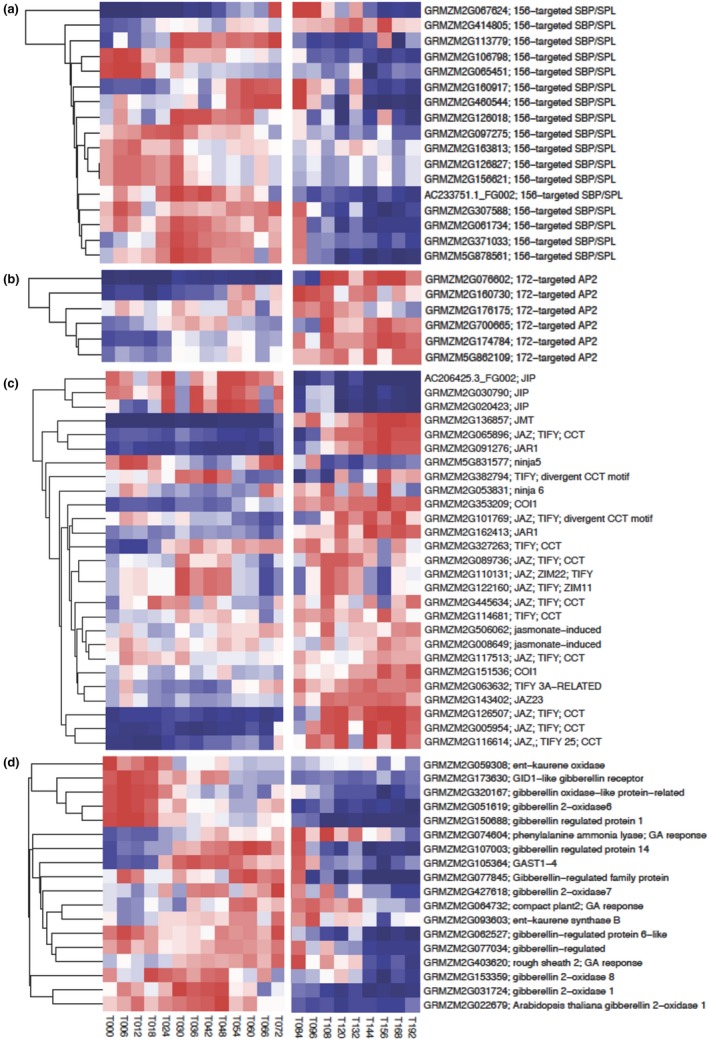
Euclidean clustering of miR156‐targeted SPB/SPLs (a), miR172‐targeted AP2 transcription factors (b), putative juvenile phase influencing JAZ transcription factor transcripts and select JA signaling genes (c), and gene involved in GA synthesis and signaling (d) from dry seed (T000) through seedling establishment (T192). Data were collected by Yu et al in 2013 every 6 hr from T000 to T072 and by Liu et al in 2015 every 12 hr from T072 to T192 (PMCID: PMC4434728), the change in timing of data points in indicated by the white vertical break in heatmap. Adult phase transcription factors, SPLs do not begin decreasing in expression until after the embryo pushes through the pericarp during germination at T072 (a). Some SPLs have high expression in the dry seed while the remaining SPLs increase in expression during imbibition. Juvenile phase transcription factors do not increase in expression until after the embryo pushes through the pericarp during germination at T084 (b). JA signaling and synthesis genes are enriched after the seed coat opens (c), however, some putative JAZ transcription factors are expressed while the seed is imbibing. Transcript for genes involved in GA synthesis and signaling are enriched during seed imbibition (d). Per row color scale with color breaks at calculated quantiles of expression. Red color indicates highest expression while blue color indicates lowest expression

The juvenile phase is under the regulation of miR156, whose expression is high early in seedling development and declines to end the juvenile phase (Beydler et al., 2016; Chuck et al., 2007; Fouracre & Poethig, 2019; Wu et al., 2009; Wu & Poethig, 2006). Weekly assays of miR156 in maize showed highest expression 7 days after planting (Chuck et al., 2007), but levels in developing and germinating embryos are unknown. The maize shoot meristem is active during embryogenesis, initiating four or five leaf primordia (which will become most if not all of the juvenile portion of the shoot after germination) before the onset of seed dormancy. To establish a chronology of phase‐specific gene regulation by miR156, we analyzed published transcriptome data spanning the dry seed stage to the emergence of the fourth leaf in whole seedlings (Liu et al., 2013; Yu et al., 2015) (PMCID: PMC4434728). During the 66 hr of imbibition before the embryo has grown enough to break the pericarp (stage T066), there are few morphological changes to the kernel; nonetheless, some 13,900 transcripts are differentially expressed (Liu et al., 2013). We examined levels of miR156 and miR172 targets (Zhang et al., 2009) to infer when regulation by miR156 begins and ends, expecting that during seedling establishment miR156‐targeted transcripts might initially be abundant but begin to decline when miR156 regulation commences. SQUAMOSA PROMOTER BINDING PROTEIN‐LIKE transcription factors (SPLs) are targeted by miR156 and promote floral induction and development of adult vegetative traits (Wu & Poethig, 2006; Wang, Czech, & Weigel, 2009; Yamaguchi et al., 2009; reviewed by Wang, 2014). We observed that two miR156‐targeted SPLs showed high transcript levels in the dry seed, and the remaining SPLs increased in expression during imbibition, peaking in expression between T030 and T084 (Figure 5a). While some miR156 targets began to decline at T066, which corresponds to embryo emergence from the pericarp, we saw a decrease in expression in the remaining miR156‐targeted transcripts after T084, a time point at which (based on external morphology) leaf one is at plastochron 8, the stage we measured the highest JA levels. At T168, 7 days after planting when miR156 levels are highest (Chuck et al., 2007), all but one miR156 target is at its lowest levels. Transcripts for miR172‐targeted AP2 transcription factors, some of which promote juvenile characteristics, increased in expression after embryo emergence and were still high at the last time point (T192, Figure 5b), which corresponds to the highest measured levels of miR156. None of these transcripts were highly expressed in the dry seed (T000, Figure 5b), and all had relatively low expression throughout imbibition although expression levels were dynamic (T006 to T060, Figure 5b). Together, these observations indicate the juvenile developmental program coordinated by miR156 begins during germination rather than earlier during embryogenesis.

Previous gene expression data had suggested that JA promotes the expression of miR156 during the juvenile phase in grasses (Beydler et al., 2016; Hibara et al., 2016); thus, JA signaling could also initiate high miR156 levels during germination. We examined the same data sets to determine when JA signaling begins. When JA levels increase, JAZ transcription factors are degraded and JA response genes are derepressed (Chini et al., 2007; Howe, Major, & Koo, 2018). Since JAZ transcription factors are transcribed during JA signaling to overcome negative effects of prolonged JA response on growth and development (Chung et al., 2010), the expression of JAZ transcription factors can be used as a marker for JA signaling. To identify candidate JAZ transcription factors in maize that may influence juvenile shoot identity, we conducted a pBLAST query against the maize genome. Putative JAZ transcription factors included in the analysis were identified by ZIM and Jas domains characteristic of transcription factors which respond to JA (Chini et al., 2007; Howe et al., 2018; Thines et al., 2007; Yan et al., 2007). Known JA signaling genes such as *JMT* and *JAR1* were also examined.

Several JA responsive genes began to increase in transcript level after the embryo ruptures the pericarp (T066, Figure 5c). A majority of JA signaling gene transcripts, including JMT and two JAR1 isoforms, significantly increase by T096 (Figure 5c), a stage that includes L1^P9^, where we had observed the highest levels of meJA (Figure 1). A subset of those JA signaling transcripts began to increase at T072, which is estimated to include L1^P4^, the earliest stage at which we measured JA. These transcript levels continued to increase in the whole seedling throughout the remaining time points in the dataset, peaking between T156 and T168, with a slight decline at the last time point (T192). Approximately one‐third of the transcripts analyzed were dynamically and robustly expressed during imbibition, although not at the maximum expression values recorded in this dataset. We also observed elevated levels of transcripts of a cluster of putative JAZ transcription factor genes and jasmonate‐induced proteins (JIPs) during imbibition between T018 and T048 (Figure 5c). Thus, JA signaling was evident at time points when levels of JA and meJA were still increasing and far below their maximum measured levels.

### 2.5 JA signaling occurs along the entire leaf blade

**Figure 6:**
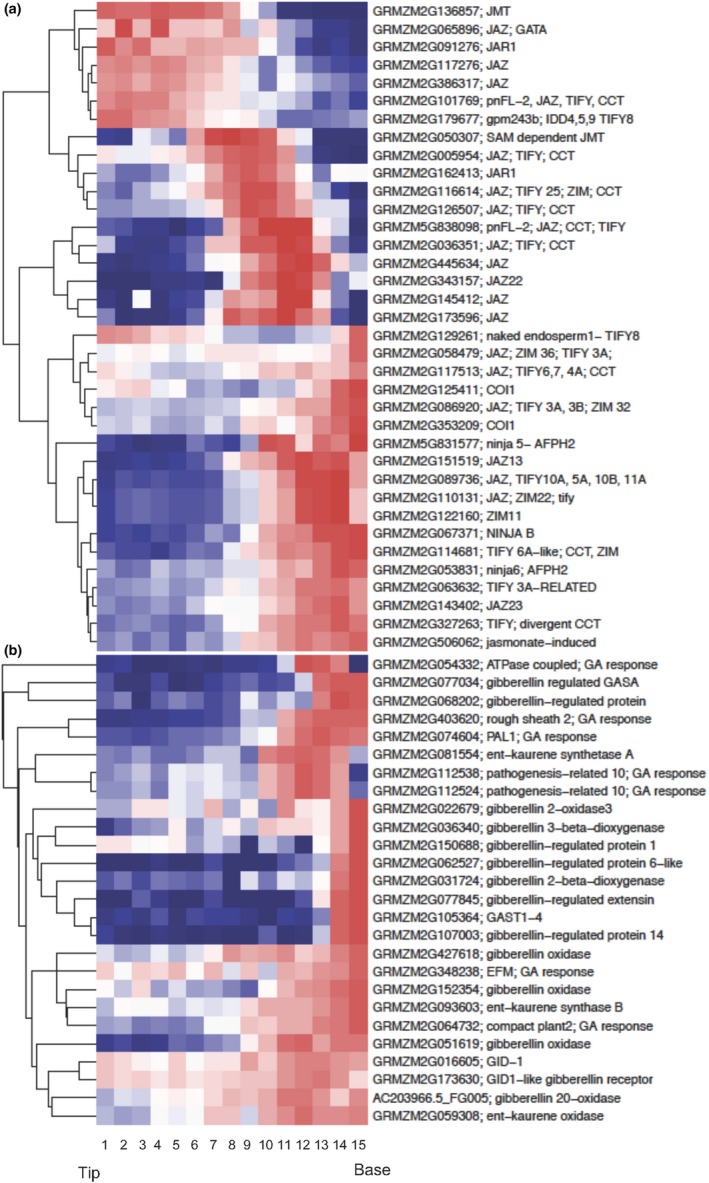
Euclidean clustering of genes involved in JA (a) and GA (b) synthesis and signaling along the leaf three blade of a 9‐day old seedling in 10 mm sections from tip (1) to base (15). These data were generated by Wang et al in 2014 (NCBI GEO series http://www.ncbi.nlm.nih.gov/geo/query/acc.cgi?acc=GSE54274). JA signaling genes are expressed throughout the leaf blade in discrete regions (a). All GA signaling genes are expressed most highly at the base of the leaf in zones 11–15 (b). Per row color scale with color breaks at calculated quantiles of expression. Red color indicates highest expression while blue color indicates lowest expression

Direct measurement of JA indicated that JA levels were not constant along the length of the blade in developing maize leaves (Figure 2). Since the previously analyzed dataset that spans seedling establishment examined whole seedlings, we were unable to determine how the variable JA levels across a juvenile leaf blade affect JA signaling. A dataset of the transcriptome of leaf three at 150 mm long (approximately at plastochron 10) in 10 mm increments generated by Wang and coworkers in 2014 (NCBI GEO series http://www.ncbi.nlm.nih.gov/geo/query/acc.cgi?acc=GSE54274) enabled us to analyze patterns in transcript levels of JA signaling genes along the leaf blade of a developing juvenile leaf. The leaf three stage (L3^P10^) captured in this dataset would correspond to T156, the time point where we saw the highest amount of JA signaling activity in the dataset generated by Liu et al and Yu et al using whole seedlings (Figure 5c). In addition, L3^P10^ contains the entire developmental gradient of maturing juvenile leaves from immature, dividing leaf tissue to mature, differentiated functional leaf tissue (Li et al., 2010). We analyzed known JA signaling genes and JAZ transcription factors as well as putative JAZ transcription factors identified using pBLAST. Transcripts of JMT, a JAR1 isoform, and a group of JAZ transcription factors were highest in the most differentiated tissue in the terminal half of leaf blade three in zones one to seven (Figure 6, top cluster). Transcripts of the SAM‐dependent JMT and a second JAR1 isoform, along with another group of JAZ transcription factors, peaked in the middle of the leaf blade in zones 6–11 (Figure 6, second cluster). Although along the leaf blade, we measured the lowest level of JA in the basal portion of both developing leaf one and seven (Figure 2), other JA signaling transcripts were high in this region of leaf three, zones 11–15 (Figure 6). Presuming L3 also has its lowest JA levels at the base, the expression of these JA signaling factors in that region indicates the reach of JA signaling extends beyond where JA levels are highest, or that the expression of those specific JA signaling genes respond to a relatively low level of JA. Together, these patterns show JA signaling genes are expressed throughout the leaf three blade in discrete groups and distinct regions of the leaf, just as JA levels varied in leaves one and seven.

### 2.6 Endogenous GA levels are higher in undifferentiated adult leaf tissue

Previous research has shown JA works synergistically (Cheng et al., 2009) with and in opposition to (Heinrich et al., 2013) GA. Additionally, GA is required for timely vegetative phase change in maize: GA‐deficient dwarf*1* mutants have a prolonged juvenile stage (Evans & Poethig, 1995). We quantified GA_3_ in leaf one at progressive developmental stages alongside JA and meJA to investigate how GA and JA levels fluctuate relative to each other during juvenile and adult vegetative development. Gibberellic acid levels in L1^P4^ to L1^P9^ were either below the threshold of detection, or, when detected, remained below 0.8 ng/g FW (Figure 1b).

We quantified GA in a developing transition and adult leaf, anticipating an increase in GA levels since previous evidence indicates that GA promotes phase change. Indeed, in transition leaf L6^P8^, GA levels were 20‐fold higher compared with L1^P6^, the leaf 1 stage having JA and meJA values similar to L6^P8^ (Figure 1, Table S2) and showing a similar degree of tissue differentiation. Gibberellic acid levels were also 20‐fold higher in developing early adult leaf L8^P10^ compared with L1^P8^ (Figure 1, Table S2). Thus, levels of GA were higher in transition and adult compared to juvenile leaves.

Gibberellic acid levels are high in the division zone at the base of expanding maize leaves compared with the remaining leaf blade (Nelissen et al., 2012), opposite of JA levels (Figure 2). To test whether variation in GA levels along the leaf blade was similar between juvenile and adult leaves, we quantified GA in the basal, middle, and tipmost 10 mm of L1^P9^ and L7^P9^. Throughout L1^P9^, GA was either below detectable levels or less than 0.5 ng/g FW. While GA levels in the tip of L7^P9^ were similar to those of L1^P9^, GA levels in the middle and base of L7^P9^ were approximately 10 and 14 times higher, respectively (Figure 2, Table S2). This is consistent with the results of Nelissen et al. (2012) who found that GA was highest at the base of developing maize leaves. In addition, measured GA levels were higher in transition leaf seven compared to juvenile leaf one at the same developmental stage (Figure 2, Table S2).

### 2.7 Phase change mutant *Teopod1* has high levels of endogenous GA

Since GA is known to promote phase change, we examined GA levels in phase change mutants, comparing mutants to their normal sibs in L1^P9^, when GA was observed at a low level, and in L6^P8^, immediately preceding phase change, when it is much higher in normal plants. As expected, GA‐deficient *d1* mutants, which have a delayed phase change, had significantly lower levels of GA compared with their normal siblings in both L1^P9^ and L6^P8^ (Figure 1). In both early adult *gl15* mutants and delayed adult *ts1* mutants, GA levels were not different between mutants and their normal sibs in L1^P9^; however, in L6^P8^ of *gl15* mutants GA levels were significantly higher than normal sibs. Gibberellic acid levels were also significantly higher compared with their normal sibs in both L1^P9^ and L6^P8^ in *Tp1* mutants: approximately 9‐fold higher in *Tp1* L1^P9^ and 80‐fold higher in *Tp1* L6^P8^ compared with normal sibs (Figure 3).

### 2.8 GA signaling is brief during germination

Gibberellic acid is well known for its role in germination; however, we detected very low levels in L1^P4^ (Figure 1), a sample comprising most of the developing shoot shortly after germination. To determine when GA signaling declines during germination, we evaluated the transcript levels of genes involved in GA synthesis and signaling from the dry seed through fourth leaf emerging (Liu et al., 2013; Yu et al., 2015) (PMCID: PMC4434728). Indeed, during seed imbibition from T000 to T084 transcripts for GA biosynthesis and signaling genes were at their highest levels, and subsequently declined after germination during seedling establishment (Figure 5d). This decline in levels of GA signaling and synthesis transcripts largely coincides with the increase in JA signaling and synthesis transcript expression (Figure 5c).

### 2.9 GA signaling is localized at the basal end of developing leaf three

We observed variation in levels of JA and GA in developing maize leaves where JA levels are higher near the tip of the leaf and GA levels are higher at the base (Figure 2). Nonetheless, various genes that mark JA signaling were expressed along the entire leaf blade in L3^P10^ (Figure 6a). We analyzed the transcript levels of GA synthesis and signaling genes in L3^P10^ in 10 mm increments (Wang et al., 2014) (NCBI GEO series http://www.ncbi.nlm.nih.gov/geo/query/acc.cgi?acc=GSE54274) to investigate whether these transcripts are expressed throughout the leaf blade or confined to regions with high GA levels. All GA signaling and synthesis transcripts used in our analysis were expressed most highly in the basal third of the developing leaf blade in zones 11–15 (Figure 6b). This indicates that, unlike JA signaling, transcripts of GA signaling genes are spatially confined to the basal division zone of the developing leaf blade that contains high levels of GA.

## 3 DISCUSSION

In this study, we quantified JA and GA in developing maize leaves to examine how endogenous hormone levels in leaves fluctuate from seedling establishment through vegetative phase change. We observed the highest level of JA and meJA in differentiated leaf tissue at the tip of leaf one, the first juvenile leaf, compared to other parts of the leaf, earlier stages of that leaf, and compared to adult or transition leaves. These direct measurements confirm the findings of a previous study that demonstrated JA signaling is upregulated in juvenile maize leaf primordia compared to adult (Beydler et al., 2016). Jasmonic acid is also required for a normal juvenile phase in rice (Hibara et al., 2016). In contrast, GA is considered to be required for the timely transition to adult as GA‐deficient mutants of both maize and rice have an extended juvenile phase (Evans & Poethig, 1995; Tanaka, 2012). Consistent with that, the highest GA levels we measured were in undifferentiated tissue that would eventually mature into adult leaf tissue. The patterns of endogenous levels of JA and GA reported here support the conclusion that high levels of JA (and low GA) maintain the juvenile phase in maize, while high GA (and low JA) is important in establishing the adult phase. The bioactive form, jasmonoyl‐isoleucine, was not assayed in this study, where we focused on the mobile meJA and its JA precursor. Although we cannot rule out variation in JA‐Ile levels being different from measured levels of JA and meJA, which did change in tandem, transcript levels for JAR1, the enzyme that converts JA to JA‐Ile, changed in lock step with those of JMT, the enzyme that methylates or demethylates JA (Figure 5c).

Previous studies revealed that juvenility could be extended by exogenously applying JA to maize seedlings, where the normal decline of miR156 was also delayed (Beydler et al., 2016). Here, we demonstrated that JA is sufficient to delay vegetative phase change and flowering but is not able to do so indefinitely. This result could be explained by the known alternative splicing of JAZ transcription factors that derepress JA target genes in *Arabidopsis*. In response to JA, JAZ transcription factors are recruited by a JA‐COI1 complex and subsequently degraded in a SCF^COI1^‐dependent manner, leading to the expression of JA response genes (Howe, 2010). To attenuate the JA response, the COI1‐interacting Jas domain of JAZ transcription factors is alternatively spliced into four progressive variants as JA signaling persists. The four splice variants of the Jas domain increasingly interact less stably with COI1 leading to unsuccessful recruitment of JAZ for degradation. This way, a plant under stress becomes increasingly less responsive to high JA levels (Chung et al., 2010; Moreno et al., 2013). Thus, it is likely JA signaling is unable to indefinitely prolong the juvenile phase because JAZ transcription factors stop responding to the hormone. After the decline in JA signaling, levels of miR156 decline, derepressing SPL transcription factors. In *Arabidopsis*, SPL9 binds JAZ transcription factors to silence a cellular response to JA, which would further negatively regulate JA signaling and facilitate the transition into the adult vegetative phase (Mao et al., 2017).

If indeed JA induces juvenility in developing maize leaves, it likely acts above some threshold level. Jasmonic acid levels in leaf primordia that would acquire juvenile traits ranged from about 0.6 to 34 ng/g, whereas levels in primordia of adult leaves were between 0.35 and 1.25 ng/g. Excluding the earliest stages of leaf one, we speculate that levels above 1.25 ng/g could be sufficient for JA signaling. The much higher level, above 30 ng/g found in L1^P8^, might reflect levels needed for production of meJA for systemic distribution. Leaf seven, as a transition leaf, has JA levels above the proposed threshold in the middle of the primordium yet most of that leaf has adult traits when expanded. However, by plastochron 9, most cell division is restricted to the basal third of the leaf (Poethig & Szymkowiak, 1996) and thus that sample will acquire juvenile traits once fully differentiated. Such a range of JA levels would be consistent with JA signaling assessed in both germinating seeds/seedlings and throughout leaf three.

During germination, a set of JA biosynthetic and signaling transcripts (including JAR1, a number of JAZ genes, and JMT) starts to increase at T084, which corresponds roughly to the stage that includes L1^P8^. At T096, when L1 is at P9, there is a substantial increase in transcript levels of those genes and a decline in miR156‐targeted SPL transcripts, consistent with a process in which increased JA signaling results in increased miR156 activity. Although there are other transcripts reflecting JA signaling that increase earlier in whole seedling development, some of these correspond to genes in barley that respond to JA present in the mature kernel to influence coleorhiza and root emergence during germination (Barrero, Talbot, White, Jacobsen, & Gubler, 2009). Supporting the focus on transcript level changes at T096 are the results of previous work comparing transcript levels in successive leaf primordia at P6 (Beydler et al., 2016). When leaf one is at P9, the stage at which we measured highest meJA, leaf four is at P6, which is the last primordium showing high levels of transcripts of a number of JIPs, two JMTs, and the most juvenile upregulated miR156s (miR156f and g).

Expression patterns of miR156 during embryogenesis and germination of maize are not known. In Arabidopsis, miR156 is involved in early embryo development (Nodine & Bartel, 2010) and expression of miR156 is high upon germination (Yang et al., 2011). By analyzing the expression patterns of miR156 and miR172 targets, we found evidence that decrease of miR156 targets and the induction of the juvenile vegetative phase do not occur until after the seed is imbibed and germination is underway. We hypothesize the stresses of germination initiate the juvenile phase in maize via JA signaling, and once the stress of seedling establishment is relieved, the plant is able to progress to the adult phase and eventually flower. This is supported by our observations that both the levels of JA, a stress signaling hormone, and transcript levels of genes involved in relieving oxidative stress are higher in juvenile leaves compared to transition and adult leaves (Figure 1; Beydler et al., 2016).

Transcripts for JA biosynthesis and signaling genes (including JMT, JAR1, and JAZs) were expressed throughout leaf three, though in distinct zones (Figure 6a). In contrast, transcripts for GA synthesis and signaling genes were restricted to the basal third of the leaf (Figure 6b). These expression patterns agree with hormone levels we measured along the leaf blade and previous research that demonstrated GA regulates cell division in the base of developing leaves (Nelissen et al., 2012). The interaction between JA and GA signaling pathways within the leaf could influence juvenile leaf morphology or size. Transcript levels for genes involved in JA synthesis and signaling decreased in each subsequent juvenile leaf at plastochron 6 (Beydler et al., 2016). The progressive increase in size of each sequential juvenile leaf correlates with the progressive decline in JA signaling. Jasmonic acid‐deficient *opr7opr8* mutants display increased juvenile leaf length (Yan et al., 2012). As maize progresses through the juvenile phase and changes to adult, levels of GA increase. Gibberellic acid signaling opposes JA signaling, impeding the inhibition of growth by JA, leading to more cell division and thus a larger leaf size. Furthermore, if JA is a systemic leaf‐derived signal influencing the phase identity of the shoot, this increase in GA signaling at the base of subsequent leaves would disrupt the promotion of juvenility by JA.

Quantification of JA and GA in vegetative phase change mutants supported the view that JA promotes juvenility in opposition to GA, which promotes the transition to adult. Biosynthetic mutants had the expected lowered level of GA (*d1*) or JA (*ts1*). In *ts1* mutants, levels of GA were not different between mutants and wild‐type sibs. In contrast, JA levels were elevated in *d1* mutants, especially in leaf 6, which in normal plants is a transition leaf but in the mutant is fully juvenile. This suggests GA signaling may negatively regulate JA levels while JA signaling does not affect GA levels. In precocious phase change mutant *gl15,* levels of JA were not different between mutants and normal sibs. This can be explained by *Gl15* being downstream of miR156 (Moose & Sisco, 1996), and miR156 levels influenced by JA (Beydler et al., 2016). While we observed increased GA associated with the onset of adult characteristics in normal plants, extended juvenile *Tp1* mutants unexpectedly showed GA levels some 80 times greater than normal. One explanation is that once increased GA levels have facilitated vegetative phase change, adult tissues are involved in a feedback mechanism that attenuates GA production, which would be missing from the prolonged juvenile phase of *Tp1* mutants. Gibberellic acid homeostasis is tightly feedback controlled by GA metabolism and signaling (Jiang & Fu, 2007): Inhibition of GA signaling by treatment with GA biosynthesis inhibitors or genetic mutation upregulates GA biosynthesis genes and downregulates GA catabolism genes (reviewed by Sun, 2010).

## 4 MATERIALS AND METHODS

### 4.1 Plant material and growth conditions

Maize inbreds B73 and Mo17, a gift from Kendall Lampkey, were crossed to generate hybrid seeds. Seeds segregating for *tasselseed1* seeds were a gift from Josh Strable. *glossy15, dwarf1,* and *Teopod1* seeds were provided by the Maize Genetics COOP Stock Center. Plants were grown in a temperature‐controlled greenhouse and illuminated for 14 hr daily under 1‐kW metal halide and sodium lights (www. osram.com). B73xMo17 hybrids were used for quantifying hormones unless otherwise stated. In this genetic background, leaves 1–4 displayed only juvenile traits, leaves 5–7 were transition leaves, with the remaining leaves displaying adult characteristics.

### 4.2 Seedling treatment and scoring

Emerging seedlings (first leaf partially expanded) received 100 μl of 5 mM JA in 5% ethanol to the apical whorl. Mock control treatments consisted of application of the same volume of solvent. Treatments with JA occurred at 2‐day intervals. Ear and tassel emergence were scored by counting leaves below the inflorescences.

### 4.3 Transcriptome analysis

The RNAseq dataset of maize dry seed to fourth leaf emerging was obtained from Yu et al., 2015. This experiment was conducted using White Crystal (Liu et al., 2013; Yu et al., 2015) (PMCID: PMC4434728), a maize cultivar that has been shown (Tausta et al., 2014) to have high similarity in transcript levels with the Mo17xB73 hybrid we employed. The RNAseq dataset of 9‐day old B73 maize leaf 3 was obtained from NCBI GEO series http://www.ncbi.nlm.nih.gov/geo/query/acc.cgi?acc=GSE54274. Heatmaps were made using the pheatmaps package in R. Transcript expression values were converted to per row color scale, and color breaks were calculated using data quantiles. Genes were clustered using complete method and Euclidean distance. Candidate genes involved in juvenile JA signaling were found using pBLAST with maize (taxid:4577) genome against refseq_protein database. Matches were scored using BLOSUM62 matrix and e‐value cutoff of 1e−5.

### 4.4 Hormone measurement

The extraction protocol was derived from Pan, Welti, and Wang (2010). Hormones were extracted from 100 mg pooled fresh leaf tissue at desired developmental time points using 2:1:0.002 v/v/v 2‐propanol:H_2_O:HCl with dihydromethyljasmonate as an internal standard (Engelberth et al., 2003; Mueller, Mène‐Saffrané, Grun, Karg, & Farmer, 2006; Ozawa, Shiojiri, Sabelis, & Takabayashi, 2008) to adjust results for extraction efficiency. Tissue samples were harvested at the same time each day to account for circadian variations in hormone levels. Extracts were purified with dichloromethane, dried, and resuspended in methanol. Hormone levels in each extract were determined using ultra‐high‐pressure liquid chromatography–tandem mass spectrometry (Acquity H‐Class UPLC—Waters TQD).

## CONFLICT OF INTEREST

The authors declare no conflict of interest associated with the work described in this manuscript.

## AUTHOR CONTRIBUTIONS

K.O. carried out experiments, conducted data analysis, and wrote the manuscript. C. L. C. consulted on experimental design and edited the manuscript. E. E. I. designed the experiments and wrote the manuscript.
