# Supplementary material for: Jasmonic acid levels decline in advance of the transition to the adult phase in maize
Source: Plant Direct. 2019 Nov 26;3(11):e00180. doi: 10.1002/pld3.180 (PMC6879778; doi:10.1002/pld3.180)

Supplementary Table 1.

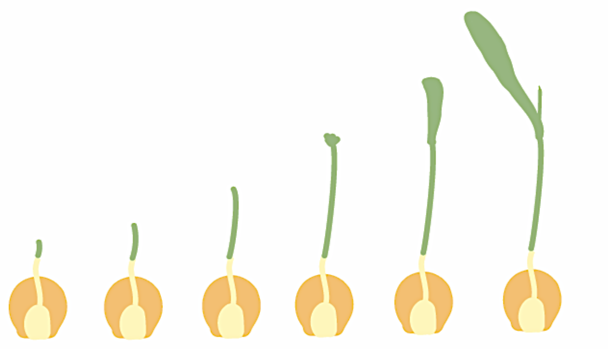

Plastochron	4	5	6	7	8	9
Leaf length (mm)	1.5 - 3	3.5 - 6	7-10	11-15	25 - 35	70 - 90

Supplementary Table 2. ng/g FW

Stage		[JA]	[meJA]	[GA]
L1	P4	0.577 ± 0.20	1.109 ± 0.46	0.440 ± 0.035
	P5	0.790 ± 0.28	1.052 ± 0.32	0.525 ± 0.33
	P6	2.873 ± 0.86	1.764 ± 0.35	0.702 ± 0.075
	P7	2.652 ± 0.23	3.138 ± 0.83	0.450 ± 0.010
	P7.5	10.614 ± 1.91	3.988 ± 1.25	0.409 ± 0.14
	P8 tip	33.895 ± 4.68	9.098 ± 1.40	0.412 ± 0.15
	P9 tip	8.833 ± 2.08	41.904 ± 6.91	0.574 ± 0.097
	P9 mid	10.500 ± 3.03	24.907 ± 1.62	0.412 ± 0.15
	P9 base	4.356 ± 1.02	1.110 ± 0.37	0.409 ± 0.14
L6	P8 tip	4.388 ± 1.30	2.850 ± 1.85	14.338 ± 5.58
L7	P9 tip	3.570 ± 1.61	5.593 ± 1.75	0.287 ± .27
	P9 mid	2.652 ± 1.30	2.250 ± 1.14	5.593 ± 3.27
	P9 base	0.348 ± 0.13	0.449 ± 0.13	7.453 ± 4.30
L8	P10 tip	1.252 ± 0.46	2.136 ± 1.23	10.426 ± 6.49

Supplementary Table 3. LC-MS Detection Peak Area of Maize Mutants

Stage	JA	meJA	GA
ts1 w.t $L1^{P9}$	421.422 ± 44.38	152.09 ± 45.65	179.21 ± 54.70
ts1 $L1^{P9}$	195.44 ± 56.07	50.14 ± 19.04	169.83 ± 62.69
gl15 w.t. $L1^{P9}$	872.67 ±118.11	374.18 ± 74.47	50.10 ± 16.99
gl15 $L1^{P9}$	856.78 ± 57.76	376.65 ± 58.87	34.88 ±3.88
gl15 w.t. $L6^{P8}$	872.39 ± 8.89	162.77 ± 43.46	40.78 ± 18.32
gl15 $L6^{P8}$	880.99 ± 50.00	163.43 ± 74.46	431.80 ± 23.14
Tp1 w.t. $L1^{P9}$	716.81 ± 167.25	232.43 ± 69.02	25.51 ± 4.78
Tp1 $L1^{P9}$	661.63 ± 86.86	187.42 ± 25.36	219.02 ± 93.36
Tp1 w.t. $L6^{P8}$	824.97 ± 63.73	176.53 ± 10.03	52.42 ± 17.52
Tp1 $L6^{P8}$	911.56 ± 231.31	245.28 ± 57.06	4163.03 ± 266.76
d1 w.t. $L1^{P9}$	450.55 ± 30.20	277.74 ± 27.62	10.22 ± 8.28
d1 $L1^{P9}$	453.87 ± 23.47	121.50 ± 34.82	2.25 ± 1.07
d1 w.t. $L6^{P8}$	508.64 ± 35.78	164.32 ± 11.65	40.77 ± 18.32
d1 $L6^{P8}$	811.41 ± 46.03	325.16 ± 128.32	10.06 ± 4.42

Supplementary Figure 1. Expression of putative DELLA transcription factors from dry seed to fourth leaf emerging.

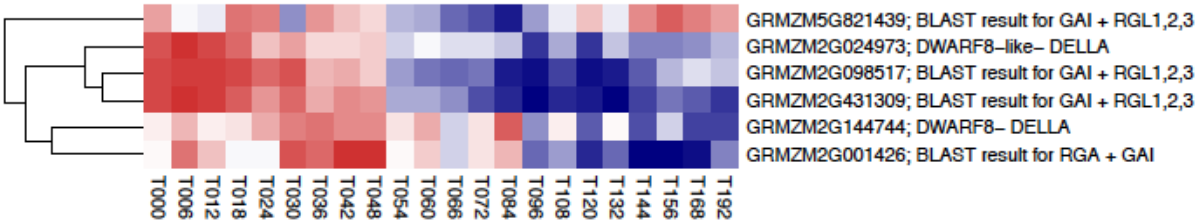

Supplementary Figure 2. Expression of putative DELLA transcription factors along the leaf blade of $L3^{P10}$.

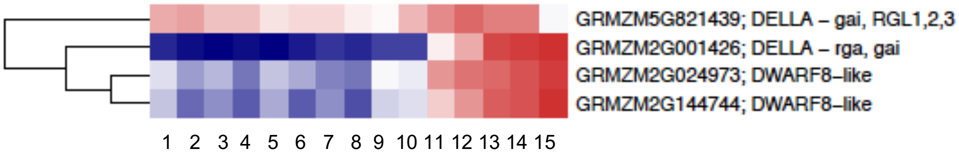

Supplement: Supplementary file 1 [file PLD3-3-e00180-s001.pdf]
